# Purification and Characterization of a Dark Red Skin Related Dimeric Polyphenol Oxidase from Huaniu Apples

**DOI:** 10.3390/foods11121790

**Published:** 2022-06-17

**Authors:** Bin Liu, Xianfang Zhou, Haiyan Guan, Xuequn Pang, Zhaoqi Zhang

**Affiliations:** 1 State Key Laboratory for Conservation and Utilization of Subtropical Agro-Bioresources/Guangdong Provincial Key Laboratory of Postharvest Science of Fruit and Vegetables/Engineering Research Center for Postharvest Technology of Horticultural Crops in South China, South China Agricultural University, Guangzhou 510642, China; bin8083@stu.scau.edu.cn (B.L.); v196057634@163.com (X.Z.); star18027249251@163.com (H.G.); xqpang@scau.edu.cn (X.P.); 2 College of Life Sciences, South China Agricultural University, Guangzhou 510642, China; 3 College of Horticulture, South China Agricultural University, Guangzhou 510642, China

**Keywords:** Huaniu apple, polyphenol oxidase, dimer, procyanidins, polymerization

## Abstract

The distinct dark-red skin of Huaniu apples renders them attractive to customers. However, the mechanism that leads to the development of the color of the fruit is unclear. In this study, we found that compared with red Fuji (a bright-red apple cultivar), Huaniu apples had higher contents of (−)-epicatechin (EC), (−)-epigallocatechin (EGC), (−)-gallocatechin gallate (GCG), and procyanidins (PCs) B2 and C1 in the peel, which implies that the polymerization of the flavanols and PCs may be correlated with the dark-red skin of the fruit. Using EC as a substrate, we purified an enzyme from Huaniu peel. We performed protein sequencing and discovered that the enzyme was a polyphenol oxidase (PPO). The molecular weight of the enzyme was approximately 140 kDa, which we estimated by native-PAGE and SDS-PAGE, while it was 61 kDa by urea-SDS-PAGE, from which we discovered that the PPO was a dimer. We observed the lowest *K*_m_ value for catechol (0.60 mM), and the best substrate was 4-methylcatechol, with a *V*_max_ of 526.32 U mg^−1^ protein. EC is a suitable natural substrate, with a *K*_m_ value of 1.17 mM, and 55.27% of the *V*_max_/*K*_m_ of 4-methylcatechol. When we used EC as a substrate, the optimum temperature and pH of the PPO were 25 °C and 5.0, respectively. In summary, we purified a dimeric PPO from Huaniu apples that showed high activity to EC, which might catalyze the polymerization of flavanols and PCs and lead to the dark-red color development of the fruit.

## 1. Introduction

Huaniu apples are a series of cultivars selected from Red Delicious apples (*Malus Domestica*, cv. Red Delicious) and grown in Gansu province, China. Compared with other apples with bright-red color, such as red Fuji, Huaniu apples have a distinct dark-red skin, which renders them attractive to customers around the world.

The red of apple skin is mainly determined by the anthocyanin content in the peel of the fruit [1,2]. During the maturation of the fruit, the anthocyanin content gradually increases, leading to the elevated degree of redness. In addition to anthocyanins, proanthocyanidins (PAs) are important compounds that contribute to the coloring of plant tissues [3,4]. PAs are the oligomers and polymers of flavan-3-ol units. Procyanidins (PCs) are the most common PAs that are constituted with (–)-epicatechin (EC) and (+)-catechin (CT) [5]. PC monomers (CT and EC) and some oligomers are colorless. After polymerization, these compounds become dark red or brown, and with a higher degree of polymerization, their colors become darker [3]. Therefore, the polymerization of mono- or oligo-PCs is one of the factors that leads to the color deepening of plant tissues. For example, in red wheat and rice seeds, the polymerization of PCs is responsible for the production of the reddish-brown pigments [4,6]. The oxidative polymerization of PCs caused the seed-coat browning of *Arabidopsis thaliana* [7]. Apple peel contains numerous PC-related substances, such as CT, EC, PC B1/B2, etc. [8,9]. The contents of these PC substances in the peels of some dark-red-skin cultivars, such as Red Delicious, are much higher than in regular red apple cultivars [10,11].

Even though the polymerization of PAs is responsible for the color deepening of plant tissues, we need to identify the enzymes that function in the polymerization in vivo. A variety of phenol oxidases, including polyphenol oxidases (PPOs) and laccases (LACs), play a role in polymerization processes due to their ability to oxidize PA monomers to quinones, which are active molecules that trigger the consequent polymerization [12,13]. For example, researchers detected oxidation polymerization products in the in vitro reaction of CT incubated with a crude PPO extract from grape [14]. Recently, we purified two laccases, LcADE/LAC and DlLAC14-4, from litchi and longan pericarp, respectively, and demonstrated their ability to catalyze the oxidative polymerization of PA monomers, such as EC and CT, and produce brown products in vitro [15,16]. The high levels of LcADE/LAC and DlLAC14-4 in litchi and longan pericarp, respectively, suggest that they play important roles in pericarp browning. In Arabidopsis, the seed coat of a *tt10* mutant, deficient in a laccase (AtLAC15/TT10), was transparent, and it contained substantially higher contents of EC and soluble PC oligomers than the wild type, which suggests that the laccase-mediated polymerization of EC and PC oligomers is responsible for the browning in vivo [7]. However, the enzymes that are responsible for the polymerization of PAs in apple peel are unknown.

In this study, to understand the mechanism that leads to the dark-red skin of apples, we first compared the contents of PA-related compounds in the fruit peels of Huaniu and Fuji, which are bright-red-skin apples. We then used EC as the substrate to purify phenol oxidases that can catalyze the polymerization of EC from the peel of Huaniu apples. We isolated a dimeric PPO that showed high activity to EC and CT. The high abundance of the PPO homodimer suggests that it may be involved in PA polymerization, which leads to the formation of the dark-red skin of apples.

## 2. Materials and Methods

### 2.1. Plant Materials

We harvested commercial-ripe Huaniu and red Fuji apples from an orchard in Tianshui, Gansu Province, China. We cleaned and peeled the fruit, kept one group of Huaniu peel frozen at −80 °C until the enzyme extraction, and we froze another group of Huaniu and red Fuji peels in liquid N_2_, and then dried the peels under the vacuum of 0.01 Pa at −80 °C for 48 h using a freeze dryer (Song Yuan freeze dryer LGJ-18, Beijing, China), then stored them at −80 °C for the extraction of flavan-3-ols and PCs.

### 2.2. Detection of the Contents of Flavan-3-ols and PCs in Huaniu and Fuji Peels by HPLC

We extracted flavan-3-ols and PCs from Huaniu and Fuji peels according to the method in our previous study [15]. We first homogenized 0.1 g of freeze-dried peel with 1.5 mL of *n*-hexane with 1% (*w/v*) butylated hydroxytoluene (BHT) and 3% (*v/v*) formic acid to remove fat-soluble substances. After we removed the *n*-hexane supernatant, the residue was homogenized with 1.5 mL of methanol with 0.2% (*v/v*) formic acid and 1% (*w/v*) BHT. We vortexed the mixture for 2 min, sonicated it at 0 ℃ for 30 min, and then centrifuged it at 10,000× *g* and 4 ℃ for 10 min. We filtered the supernatant via a 22 µm thick polyvinylidene difluoride membrane (ANPEL Scientific Instruments, Shanghai, China). We re-extracted the residue by the methanol solution following the above-mentioned procedure and combined the supernatants of the two methanol extracts.

We analyzed the contents of flavan-3-ols and PCs in the above methanol extract from red Fuji and Huaniu peels by a high-performance liquid chromatography (HPLC) system (Agilent 1260 Infinity Ⅱ, USA). We performed the HPLC analysis by using an Agilent Poroshell 120 EC-C18 column (2.7 μm, 150 × 4.6 mm) for the reversed-phase separation. We used methanol (A) and 0.2% (*v/v*) aqueous formic acid (B) for the mobile phase by applying the programmed gradient as follows: 0–15 min, 17–20.8% A in B; 15–28 min, 20.8–21.1% A in B; 28–32 min, 21.1–25.7% A in B; 32–45.2 min, 25.7–27.1% A in B; 45.2–60 min, 27.1–31% A in B; 60–63.7 min, 31–33.5% A in B; 63.7–70 min, 33.5–53% A in B; 70–75 min, 53–17% A in B; and 75–80 min, 17% A in B. The injection volume was 10 μL for all samples. The flow rate was 0.5 mL min^−1^ for a total run time of 80 min. We maintained the column at 30 ℃ and recorded the monitoring wavelength at 280 nm. We identified flavan-3-ol and PC compounds by a comparison to the retention times of the standards, including EC, CT, (+)-gallocatechin (GC), (−)-epigallocatechin (EGC), (−)-epigallocatechin gallate (EGCG), (−)-gallocatechin gallate (GCG), (−)-epicatechin gallate (ECG), (−)-catechin gallate (CG), gallic acid (GA), and procyanidins (PCs) B1, A2, B2, and C1. We calculated the contents of the identified compounds in the extraction on the basis of the standard curves (Figure S1).

### 2.3. Anthocyanin-Content Determination

We performed the anthocyanin-content determination as described by Fang et al. [17]. We immersed 1 g of freeze-dried apple peel in 60 mL of 0.1 M HCl in water, and the supernatant was collected. The residues were re-extracted 3 times with 20 mL of 0.1 M HCl each until the extraction was colorless. The supernatant of the 4 extractions were combined and brought to 120 mL. We used a pH-differential method for the anthocyanin-concentration determination [18]. We diluted the extract of 5 mL in 25 mL of 0.4 M pH 1.0 KCl–HCl buffer, and in 25 mL of 0.4 M pH 4.5 citric acid–Na_2_HPO_4_ buffer. Absorbance of the dilutions at 510 nm and at 700 nm was determined by a spectrophotometer (Shimadzu UV-2450, Japan). The absorbance at 700 nm (haze) was subtracted from that at 510 nm to get a corrected absorbance (A). Difference in the corrected absorbance (ΔA) at 510 nm between pH 1.0 and pH 4.5 dilution was determined. Anthocyanin content (as cyanidin-3-glucoside) was calculated by the method of Wrolstad et al. [18]:

Anthocyanin content (mg^−1^ g^−1^ DW) = ΔA × L × V_1_ × MW/ε × V_2_/m (1)

L (cuvette thickness) = 1 cm; V_1_ (the diluted extract) = 5 mL; V_2_ (the total extract) = 120 mL; m (peel) = 1 g. For cyanidin-3-glucoside, ε = 29,600 mol^−1^ cm^−1^ and MW = 445 g mol^−1^.

### 2.4. Enzyme Extraction and Purification

The enzyme extraction and purification were carried out using the method of Han et al. [19] and our previous study [15]. We ground 200 g of frozen Huaniu peel to powder by liquid N_2_ and added it to 600 mL extraction buffer (0.05 M pH 7.0 phosphate buffer (PBS), containing 50% (*w/v*) polyvinyl-polypyrrolidon (PVPP) and 1% (*w*/*v*) protease-inhibitor cocktail (Sigma-Aldrich, Merck, NJ, USA)). After centrifugation at 13,000× *g* and 4 °C for 30 min, we collected the supernatant as a crude protein solution. We first fractionated the crude protein solution by precipitation with ammonium sulfate from 25% to 75% saturation and centrifugation at 13,000× *g* and 4 °C for 20 min. We dissolved the pellet in the PBS buffer and dialyzed it against the same buffer overnight. We loaded the dialyzed solution onto a DEAE-sepharose column that we had equilibrated with the PBS buffer. We eluted the enzyme from the column at a flow rate of 0.3 mL min^−1^ with a NaCl linear gradient (0–0.5 M) in the PBS buffer. We collected and determined fractions of 1 mL for the enzyme activity and protein contents (A280). We combined and concentrated the fractions with high activity by an ultrafiltration centrifuge tube (Amicon Ultra-15 Centrifugal Filter Units, Merck KGaA, Darmstadt, Germany). We further loaded the concentrated fraction onto a Sephadex G-200 column that we equilibrated and eluted with the PBS buffer. We combined the fractions with high ratios of activity-to-protein content as the purified enzyme for the following analysis. EC was the substrate of the enzyme activity assay in the purification process.

### 2.5. Electrophoresis Study, Molecular-Weight Estimation, and Sequencing

According to our previous study [15], we performed the activity staining of the purified proteins on 10% (*w*/*v*) sodium dodecyl sulfate–polyacrylamide gel electrophoresis (SDS-PAGE), without boiling the protein sample. We rinsed the gel twice with a citrate phosphate buffer (50 mM, pH 6.0) for 5 min each time for the removal of the SDS. Brown bands were visible after we immersed the gel in the same buffer with 5 mM EC for 5 min.

We denatured the purified proteins by boiling in Laemmli’s sample buffer for 10 min, and then separated them by using 10% (*w*/*v*) SDS-PAGE (with or without 8 M urea), at a constant current of 110 V [20]. We stained the proteins with Coomassie Brilliant Blue R-250 (Sigma-Aldrich). We estimated the molecular weight by comparison to protein marker PM2510 (SMOBIO Technology, Inc., Beijing, China). We excised the bands of SDS-PAGE and urea-SDS-PAGE, and protein sequencing was performed by Sangon Biotech (Shanghai, China) via Biolynx peptide sequencing.

### 2.6. Activity Assay of PPO

The enzymatic activity was determined using the spectrophotometric procedure by Liu et al. [15] and Helga et al. [21] with some modifications. In this experiment, we mixed 1 mL pH 5.0 citrate phosphate buffer and 5 mM EC with 0.005 mL of the purified enzyme fraction. We incubated the reaction in triplicate at 25 °C for 10 min, and we terminated it by adding 1 mL of chloroform. We determined the activity in a spectrophotometer at 398 nm (A398). We defined one unit of enzyme activity as a 0.01-unit change in the absorbance per minute.

### 2.7. Effect of Temperature and pH on Enzymatic Activity

To establish the optimum temperature and pH of the purified enzyme, we subjected the enzyme to an activity assay at different temperatures ranging from 5 to 55 °C and pH ranging from 2.5 to 8.0. To determine the thermal stability of the enzyme, we first incubated the purified enzyme in a thermostatic water bath at various temperatures ranging from 5 to 55 °C for 24 h, and we then assayed the activity. Moreover, to determine the pH stability, we maintained the purified enzyme in a range from 2.5 to 8.0 in 0.1 M citrate phosphate buffers for 24 h, and we then determined the activity. We estimated the relative activity as a percentage by comparing it to the highest activity.

### 2.8. Substrate Specificity and Kinetic Parameters

We determined the substrate specificity of the purified enzyme under the optimized pH and temperature conditions using five substrates: 4-methylcatechol, catechol, EC, CT, and chlorogenic acids. We estimated the Michaelis–Menten constant (*K*_m_) and maximum catalytic velocity (*V*_max_) for each substrate by using Lineweaver–Burk plots (Figure S2), according to Liu et al. [15].

### 2.9. Statistics Analysis

We acquired the means ± standard errors (SEs) (*n* = 3) with three replicates. We calculated the differences between the samples by Student’s *t*-test by applying SPSS (IBM Inc. 22, Armonk, NY, USA) statistics at the 99% and 95% confidence levels.

## 3. Results and Discussion

### 3.1. Contents of Flavan-3-ols, PCs, and Anthocyanins in Huaniu and Red Fuji Apple Peels

**Figure 1:**
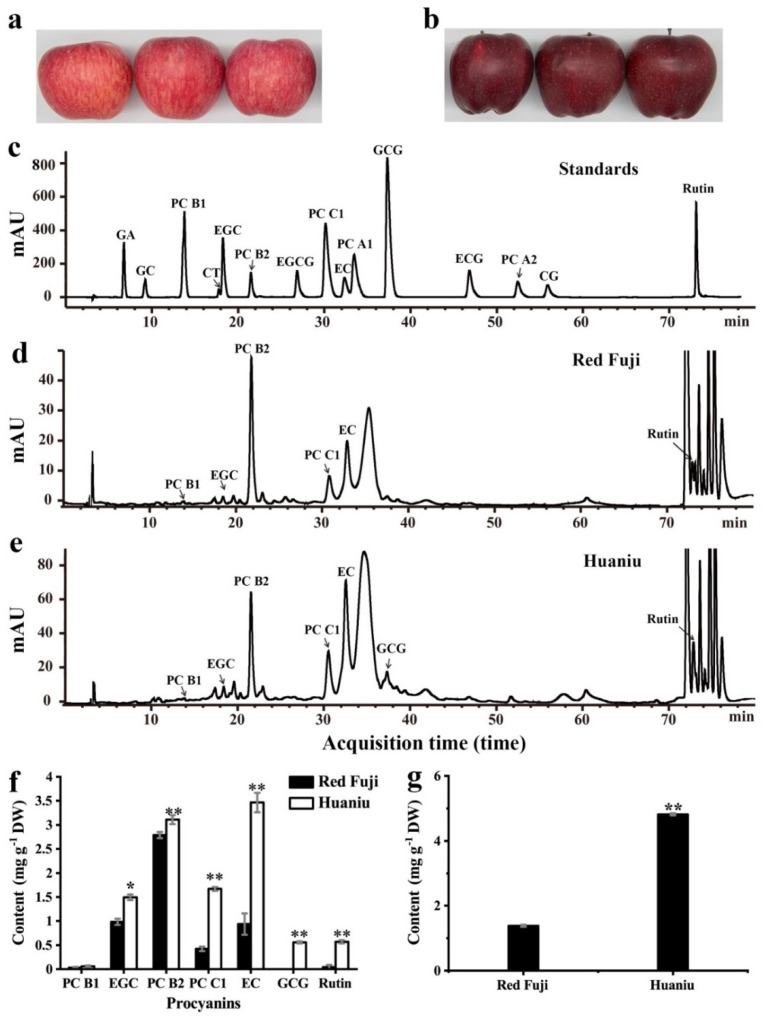
Contents of flavan-3-ols, procyanidins (PCs) and anthocyanins in red Fuji and Huaniu peels. (**a**,**b**) Images of (**a**) red Fuji and (**b**) Huaniu apples. (**c**) HPLC chromatogram of standards of flavan-3-ols and PCs. (**d**,**e**) HPLC chromatogram of flavan-3-ols and PCs extracted from (**d**) red Fuji and (**e**) Huaniu peels. (**f**) Contents of flavan-3-ols and PCs in Huaniu and red Fuji peels. (**e**) Contents of anthocyanins in Huaniu and red Fuji peels. Values are means of three biological repeats. Error bars indicate standard errors of means (SEMs) of the values. Asterisks indicate significant differences between Huaniu and red Fuji peels by Student’s t-test (* *p* < 0.05; ** *p* < 0.01). GA: gallic acid; GC: (+)-gallocatechin; CT: (+)-catechin; EGC: (−)-epigallocatechin; EGCG: (−)-epigallocatechin gallate; EC: (−)-epicatechin; GCG: (−)-gallocatechin gallate; ECG: (−)-epicatechin gallate; CG: (−)-catechin gallate; PC: procyanidin.

We determined the contents of flavan-3-ols and PCs in the peels of Huaniu and red Fuji by HPLC. Compared to the retention times of the standards (including 10 falvan-3-ol monomers, 4 dimers, and 1 trimer), we detected EGC, EC, rutin, PC B1, PC B2, and PC C1 in red Fuji and Huaniu peels; we only detected GCG in the Huaniu peel (Figure 1c–e). This is consistent with several previous studies where flavanols (EC, CT, dimeric, oligomeric and PCs) were the most abundant phenolic compounds presented in apple peel [10,22]. In this study, the content of PC B2 was the highest in red Fuji peel, followed by those of EGC and EC, with values of 2.79 ± 0.06, 0.99 ± 0.06, and 0.94 ± 0.22 mg g^−1^ DW, respectively. EC had the highest content in Huaniu peel, followed by PC B2 and PC C1, with values of 3.47 ± 0.2, 3.11 ± 0.09, and 1.67 ± 0.04 mg g^−1^ DW, respectively. The contents of most flavan-3-ols and PCs in Huaniu were significantly higher than those in red Fuji (Figure 1f). We detected around 4.81 ± 0.03 mg g^−1^ DW of anthocyanins in Huaniu peel, which was four-fold higher than that in red Fuji (Figure 1a,b,g). Our results are consistent with previous studies that the main flavan-3-ols and PCs detected in apples were EC, CT, PC B1 and PC B2, among which PC B2 and EC had relatively higher contents [8,23,24]. Similar to Red Delicious, EC was the most abundant flavanol substance in Huaniu [8,24].

Anthocyanins are present in the cultivars of red or partially red apples, and the degree of the redness is positively correlated with the anthocyanin content [8,10,22]. The anthocyanin content in the Huaniu peel was four-fold higher than that in the Fuji peel (Figure 1g); however, the Fuji apples showed a bright-red color, while the Huaniu apples showed a dark-red color, which suggests that the color of apple skin is not only determined by the anthocyanin contents, but also by other substances that may be involved. Here, we detected that the contents of flavanols (EC and EGC) and two PCs (PC B2/C1) in Huaniu peels were significantly higher than those in red Fuji peels (Figure 1f). Red Delicious apples also have 10-fold higher flavanol and PC contents in their peel than Empire apples. Even though these two apple cultivars have similar levels of anthocyanins (148.9 and 208.2 µg g^−1^ DW respectively), Red Delicious apples are dark-red, while Empire apples are bright red [8]. Based on these results and the data in the present study, we assume that the dark-red color of apples is probably due to the high contents of flavan-3-ols and PCs in the peel of Huaniu and Red Delicious.RDRD Among the others, EC and EGC are the preferred substrates for litchi laccase (LcADE/LAC) and longan laccase (DlLAC14-4), and are prone to polymerization by these enzymes [15,16]. The oxidative polymerization of flavanols and PCs was demonstrated to be closely related to the seed coat darkening of *Arabidopsis thaliana* [7]. Therefore, we suspected that the oxidation polymerization of the abundant flavanol and PC substances, catalyzed by certain phenol oxidases, might contribute to the dark skin of Huaniu apples.

### 3.2. Purification of Phenol Oxidase from Huaniu Peel with EC as Substrate

**Figure 2:**
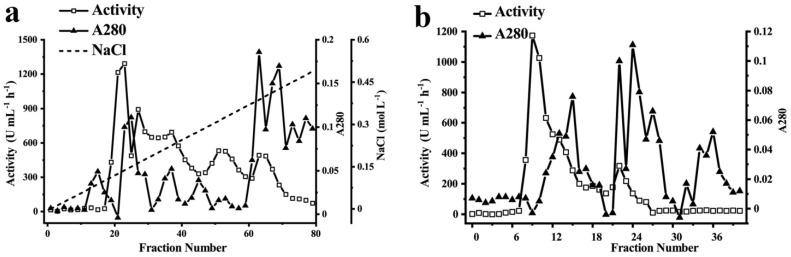
Purification of polyphenol oxidases from Huaniu peel. (**a**) Protein content (A280 nm) and activity determined in DEAE-sepharose column-chromatography fractions. Linear increase in NaCl concentration (0–0.6 M) during elution is also shown. We collected the major activity fractions for further purification. (**b**) Total protein (280 nm) and activity determined in Sephadex G-200 column-chromatography fractions. We collected the fractions in the major activity peaks for the following analysis. (**a**,**b**) We determined the activity in the fraction with EC as substrate.

**Figure 3:**
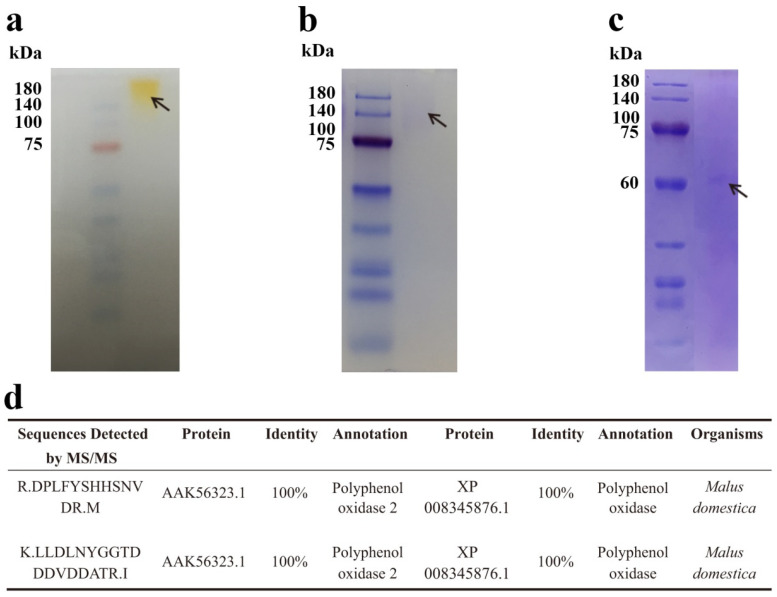
Electrophoresis analysis and sequencing of purified PPO from Huaniu peel. (**a**) Native-PAGE, (**b**) SDS-PAGE, and (**c**) urea-SDS-PAGE of purified faction after 3 steps of purification, as described in Table 1. (**d**) We detected fragment sequences identical to two apple PPOs in the bands of (**b**) SDS-PAGE and (**c**) urea-SDS-PAGE by tandem mass spectrometry (MS/MS).

We purified the phenol oxidases from the Huaniu peel by ammonium sulfate precipitation, ion-exchange chromatography, and gel-filtration chromatography. We used EC as the substrate to determine the enzymatic activity of the fractions. We applied the fractions after ammonium sulfate precipitation to DEAE-sepharose anion-exchange chromatography (Figure 2a). After elution, we determined three activity peaks. We combined the fractions of the major activity peak (Tube No. 19–25), concentrated by an ultrafiltration centrifuge tube, and then applied them to a Sephadex G-200 gel column (Figure 2b). After elution, we detected one major activity peak (Tube No. 8–14). We combined, concentrated, and applied the fractions of the peak to separation by native-PAGE, SDS-PAGE, and urea-SDS-PAGE gels. We observed a single band of protein in each gel after separation (Figure 3).

In summary, we purified a phenol oxidase that was active to EC from Huaniu peel 17.21-fold, with a specific activity of 3012.5 U mg^−1^ protein, by using the above-described three steps of purification (Table 1). The total enzyme activity considerably decreased after ammonium sulfate precipitation, whereas it showed relatively less of a decrease after the ion-exchange chromatography and gel-filtration chromatography. In contrast with the total enzymatic activity, the specific activity gradually increased with the program of the purification, which indicated the increase in the purity of the enzyme.

### 3.3. Electrophoresis, MW Determination, and Sequencing of Purified Phenol Oxidase

On the basis of the results of the native-PAGE, SDS-PAGE, and urea-SDS-PAGE, the purified enzyme after the three steps of purification appeared to be a homogenous protein with a single band in the respective gels (Figure 3a–c). The visible bands in native-PAGE and SDS-PAGE showed that the molecular weight (MW) of the purified enzyme was about 140 kDa. However, when the protein was loaded onto the urea-SDS-PAGE, which is a more denatured PAGE than SDS-PAGE, we observed a single band of about 61 kDa. The results indicate that the enzyme might be a homodimer with a 61 kDa weighted monomer. We cut and sent the bands in SDS-PAGE and urea-SDS-PAGE for tandem mass spectrometry (MS/MS) protein sequencing. The fragment sequences detected in the bands of SDS-PAGE and urea-SDS-PAGE were the same, which further confirmed that the native state of the enzyme is a dimer. We identified two peptides with high degrees of similarity to published protein sequences (Figure 3d). The sequence of the first peptide of 15 amino acids showed 100% identity to the protein sequences of *Malus domestica* polyphenol oxidase 2 (PPO2, AAK56323.1) and polyphenol oxidase (PPO, XP 008345876.1). The second peptide, with 20 amino acids, also showed 100% identity to the two PPO sequences. On the basis of the result that two peptides identified by MS/MS showed high identities to the protein sequences of PPOs from *M. domestica*, we assumed that the purified enzyme was a dimeric PPO.

As described above, we extracted the enzyme without the addition of detergents (Table 1), which indicated that it was a soluble PPO (sPPO). Researchers have also purified dimeric sPPOs with apparent MWs of 58–62 kDa from Granny Smith [19] and Goden Delicious apples [25]. Although the dimeric sPPO from Huaniu apples showed different MWs than these dimeric sPPOs, we observed dimeric PPOs in various cultivars of apples. The authors of one study purified a dimeric sPPO from soursop (*Annona muricata*) with a molecular weight of 112 kDa, which is similar to the MW of the dimeric PPO in the present study [26]. Furthermore, researchers have found dimeric PPOs in some other fruit, such as mango (*Mangifera indica*) [27] and jackfruit (*Artocarpus heterophyllus*) [28], which indicates that PPO dimers might be common in fruit and might have a specific function that is different from the monomers.

### 3.4. Substrate Specificity of Dimeric PPO

PPOs catalyze the oxidation of various phenolic substrates, and usually mono- and diphenols; however, the affinity for each compound depends on the enzyme sources. To determine the substrate specificity of the dimeric PPO purified from Huaniu peel, we investigated 4-methylcatechol, catechol, EC, chlorogenic acid, and CT. Regarding the substrate affinity, according to the *K*_m_ values, the enzyme presented the highest affinity to catechol. We observed the lowest *K*_m_ for catechol (0.60 mM), followed by EC (1.16 mM) and chlorogenic acid (1.29 mM). The substrates suitable for an enzyme can be judged by the highest catalytic efficiency (*V*_max/_*K*_m_). In this regard, the *V*_max_/*K*_m_ (U mg^−1^ protein mM^−1^) ratio for 4-methylcatechol was 212.77, followed by 140.07 for catechol and 119.05 for EC (Table 2). Accordingly, 4-methylcatechol appeared to be the most suitable substrate for the dimeric PPO. Researchers have also reported that 4-methylcatechol [29] and catechol [30] are the most suitable substrates for PPO purified from apples. Moreover, for the PPOs purified from round brinjal (*Solanum melongena*) [31], royal dates (*Ziziphus jujuba*) [32], lily bulb (*Lilium spp*) [33], and peach (*Prunus persica*) [34]' 4-methylcatechol is the most suitable substrate. Flavanols (mainly EC) and PCs are the most abundant phenolic substances in apples [8,10]. Because 4-methylcatechol and catechol were not found in apples [8], and the *V*_max_/*K*_m_ value of the enzyme on EC is 55.27% of its value on 4-methylcatechol, we suggest that EC is a relatively suitable natural substrate for the dimeric PPO from Huaniu. The relative activity of the dimeric PPO on EC to 4-methylcatechol appeared to be higher than that of the two apple PPO monomers (the relative activity on EC to 4-methylcatechol was around 10%) [20], which was probably due to the enzymatic property change between the dimers and monomers. The preferred substrate of a PPO purified from lotus seeds is EC [35], and that the enzyme can catalyze the polymerization of oligomer PCs to form hyper-polymers of PCs, which might lead to the browning of lotus seed coats [36].

### 3.5. pH and Temperature Optima of the Dimeric PPO

**Figure 4:**
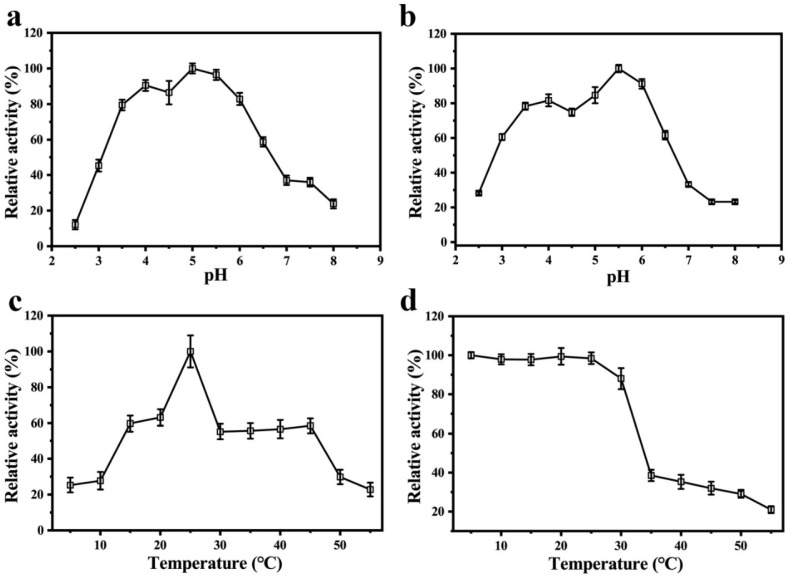
Enzymatic properties of purified PPO from Huaniu peel: (**a**) effect of pH on enzyme activity; (**b**) acid–base stability of PPO; (**c**) effect of temperature on enzyme activity; (**d**) thermal stability of PPO.

We studied the influence of pH on the PPO by using EC as a substrate for a wide range of pH values (2.5–8.0). The optimum pH value that corresponded to the maximum activity was 5.0 (Figure 4a). The results of both the optima pH and acid–base stability showed that the relative enzyme activity of the PPO could maintain more than 60% of the highest enzyme activity when the pH range was 3.5 to 6.0. When the pH values were beyond this range, the relative activity of the enzyme rapidly decreased (Figure 4b), which suggests that the dimeric PPO is more active and stable in slightly acidic environments. PPOs from fruit are more active in neutral or slightly acidic environments. For example, the optimum pH values for the PPOs from the peels of snake fruit [37] and Granny Smith apples [38] are 6.5 and 7.0, respectively.

When we used EC as a substrate, the optimum temperature of the dimeric PPO from the Huaniu peel was 25 ℃ (Figure 4c). The results of thermal stability analysis showed that the activity of the dimeric PPO remained above 80% in the range of 5–30 ℃, while it rapidly decreased when the temperature was above 35 ℃ (Figure 4d), which indicates that the dimeric PPO is a thermally sensitive enzyme. A PPO monomer that was purified from snake fruit was also sensitive to high temperature, with a notable decrease in the enzyme activity when the temperature was higher than 30 ℃ [35]. Researchers have studied the activity dependence on temperatures in PPOs from different fruits [24]. In the case of apples, a PPO from red Fuji fresh showed the highest activity at 45 and 55 °C, using catechol as a substrate [39]; however, in Golden Delicious [25] and Granny Smith apples [39], the highest activity was observed at 35 °C when using the same substrate. The optimum temperatures for the activity of PPOs usually depend on the plant species, cultivar, and tissue. However, it may also be affected by the type of substrate that is used in the activity assay [40].

## 4. Conclusions

We detected that the contents of flavanols and PCs (particularly EC) in Huaniu peel were substantially higher than in red Fuji, which might be closely related to the dark-red skin of Huaniu apples. We purified a 140 kDa dimeric PPO from Huaniu peel. The optimum temperature and pH of the enzyme were 25 °C and 5.0, respectively, and 4-methylcatechol was the most suitable substrate, whereas EC was a suitable natural substrate. The high activity of the dimeric PPO to EC may be responsible for the development of the dark-red skin of Huaniu apples.

## Figures and Tables

**Table 1:** Purification of polyphenol oxidase from Huaniu peel.

Purification Step	Total Protein (mg)	Total Activity (U)	Specific Activity (U mg^−1^ Protein)	Purification (Fold)	Yield (%)
PBS extract	1204.97	203,544	168.92	1.00	100.00
(NH_4_)_2_SO_4_ ^a^	18.40	8895	483.42	2.86	4.37
DEAE-sepharose ^b^	4.49	3429	763.70	4.35	1.62
Sephadex G-200 ^c^	0.40	1205	3012.5	17.21	0.57

^a^ Saturations of 25% and 70% of (NH_4_)_2_SO_4_ were used for sequential precipitation of protein extract. ^b^ DEAE: Diethylaminoethyl (DEAE)-sepharose column chromatography. ^c^ Sephadex G-200: Sephadex G-200 column chromatography.

**Table 2:** Substrate specificity of dimeric PPO from Huaniu peel.

Substrate	*K_m_* (mM)	*V*_max_ (U mg^−1^ Protein)	*V*_max_/*K*_m_ (U mg^−1^ Protein mM^−1^)	% *V*_max_/*K*_m_
4-methylcatechol	2.47	526.32	212.77	100.00
Catechol	0.61	85.00	140.07	65.83
EC	1.17	138.89	119.05	55.95
Chlorogenic acids	2.22	124.95	56.30	26.46
CT	4.63	85.47	18.44	8.67

## Data Availability

The data presented in this study are available on request from the corresponding author.

## Supplementary Materials

The following supporting information can be downloaded at: https://www.mdpi.com/article/10.3390/foods11121790/s1, Figure S1: The standard curves of the flavan-3-ol and PC compounds; Figure S2: Line weaver-Burk plots for kinetic parameter measurement of the dimeric PPO from Huaniu peel.
