# Supplementary material for: Purification and Characterization of a Dark Red Skin Related Dimeric Polyphenol Oxidase from Huaniu Apples
Source: Foods. 2022 Jun 17;11(12):1790. doi: 10.3390/foods11121790 (PMC9223062; doi:10.3390/foods11121790)
Supplement: Supplementary file 1 [file foods-11-01790-s001.zip › foods-1757188-supplementary.pdf]

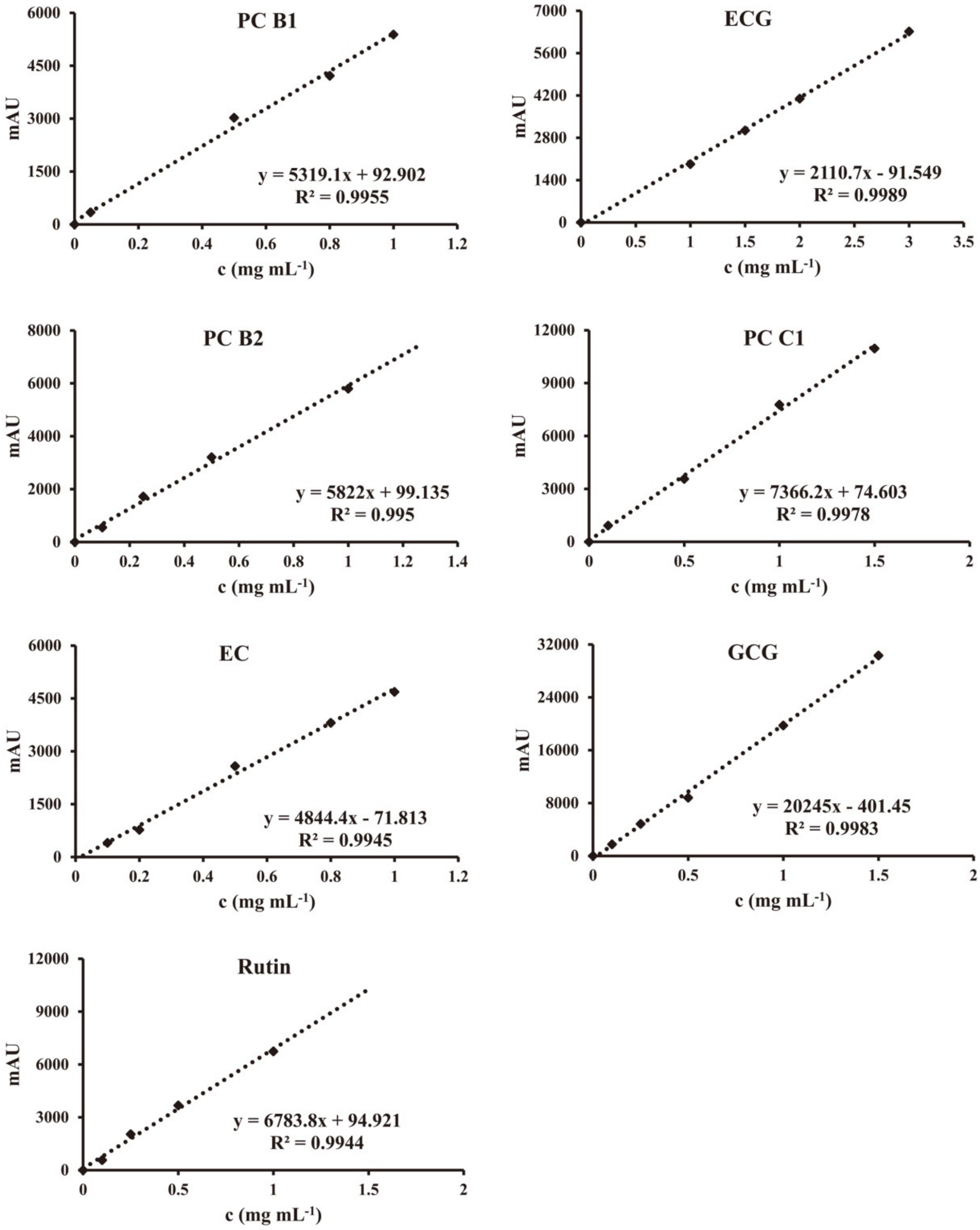

Figure S1. The standard curves of the flavan-3-ol and PC compounds.

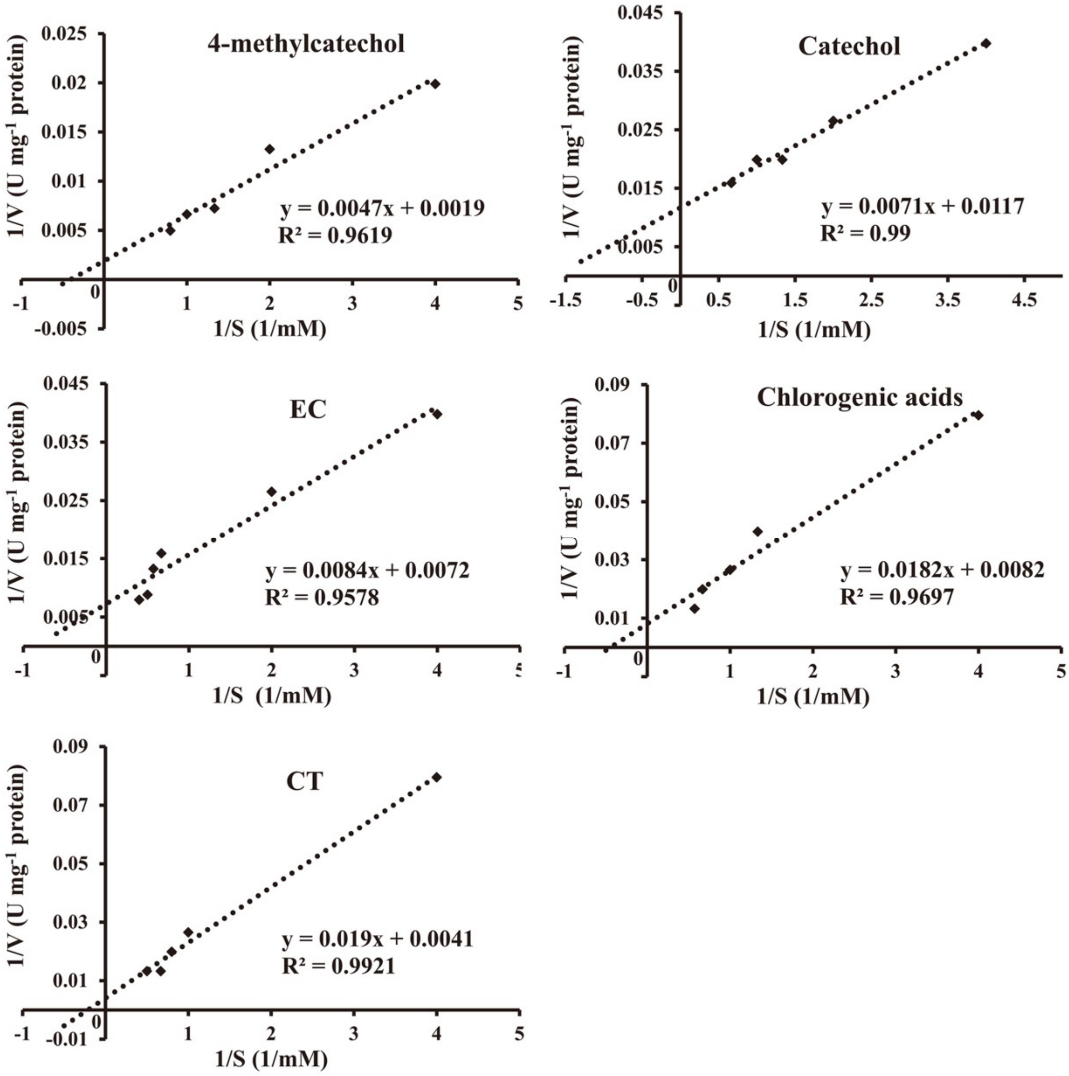

Figure S2. Line weaver-Burk plots for kinetic parameter measurement of the dimeric PPO from Huaniu peel.
